# Supplementary material for: Response of carbon cycle to drier conditions in the mid-Holocene in central China
Source: Nat Commun. 2018 Apr 10;9:1369. doi: 10.1038/s41467-018-03804-w (PMC5893629; doi:10.1038/s41467-018-03804-w)
Supplement: Supplementary file 1 — Supplementary Information [file 41467_2018_3804_MOESM1_ESM.pdf]

Supplementary Information

Title: Response of Carbon Cycle to Drier Conditions in the Mid-Holocene in Central China

Huang et al.

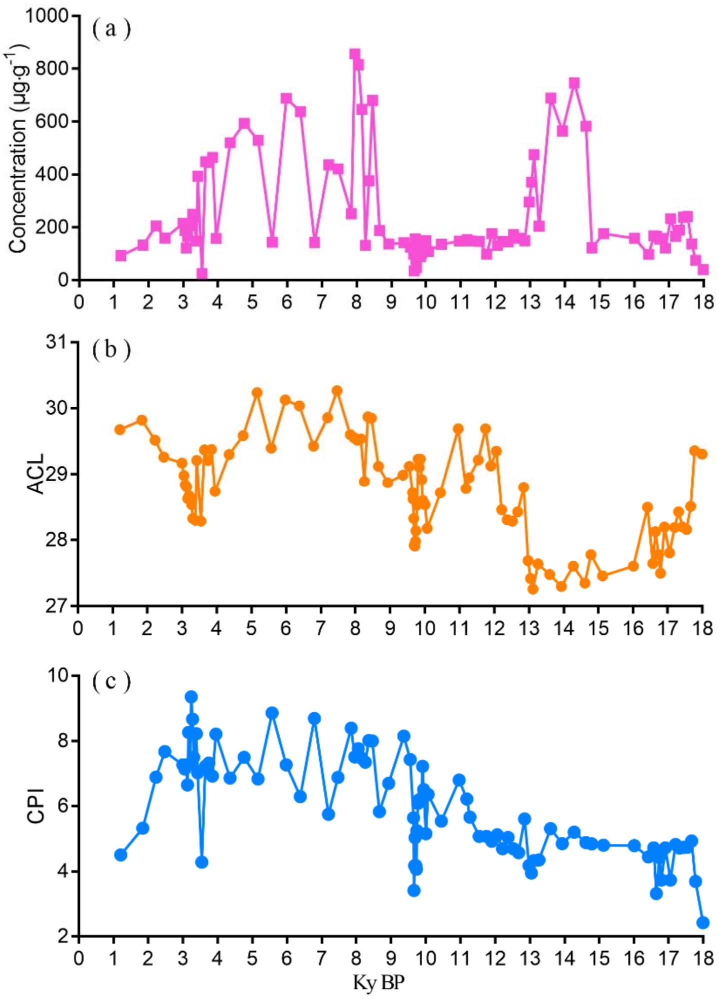

Supplementary Figure 1. Plots of *n*-alkane concentration and ratios in ZK-5 core. (a) Total alkane concentration. (b) Average chain length (ACL). (c) Carbon preference index (CPI).

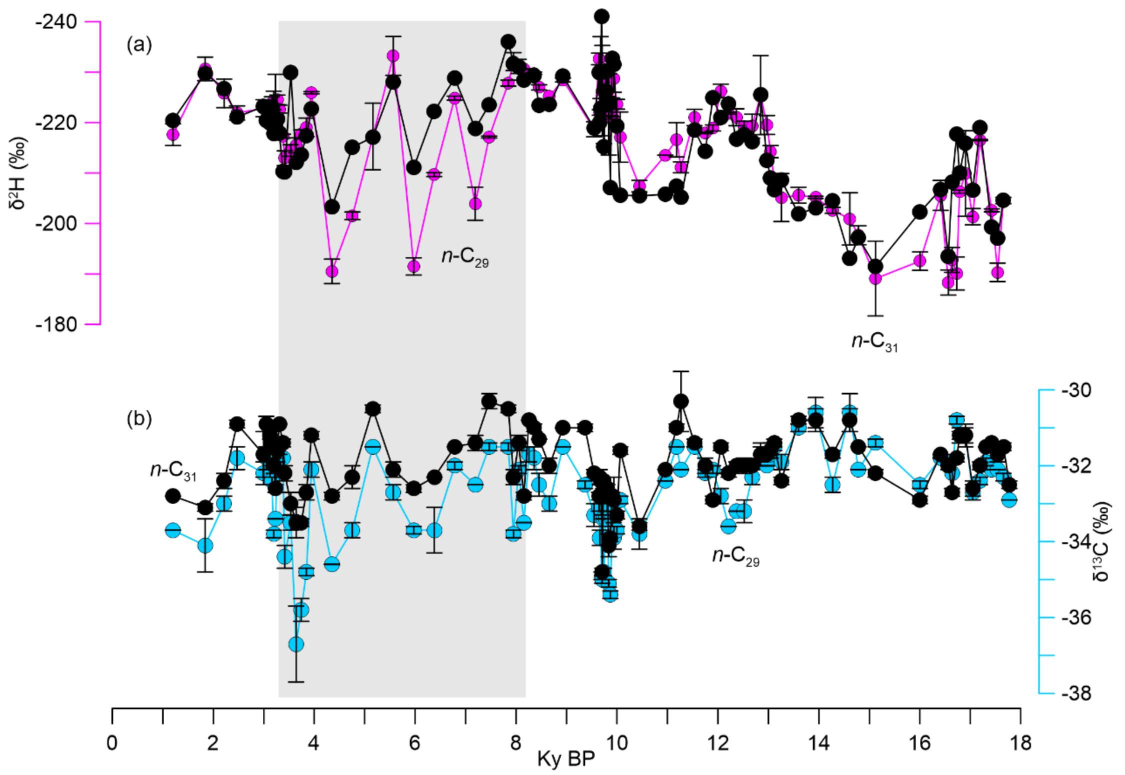

Supplementary Figure 2. Plots of the δ^2H and δ^{13}C values of C_{29} and C_{31} *n*-alkanes in ZK-5 core. (a) **δ^2**H and (b) **δ^{13}**C values of C_{29} and C_{31} *n*-alkanes. Error bars represent 1 s.d. of replicate runs. The grey shading infers the drier interval in the mid-Holocene.

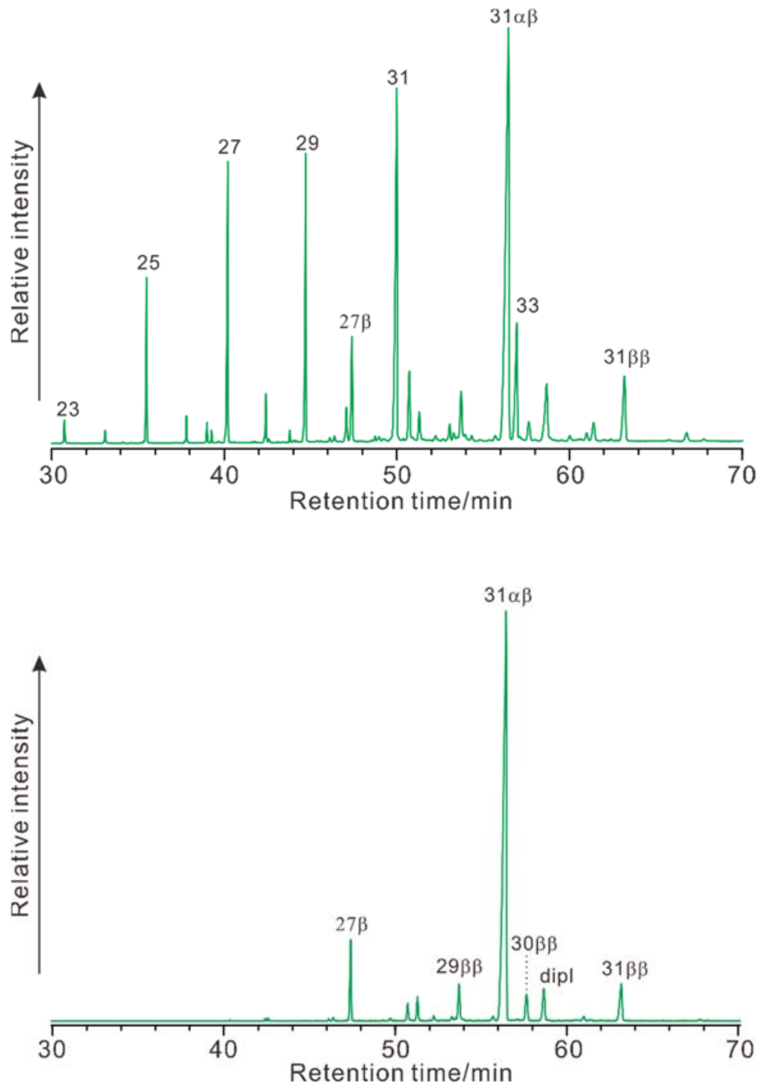

Supplementary Figure 3. Chromatograms of hopanes in a selected sample from ZK-5 core. (a) Total ion chromatogram (TIC). (b) *M/z* 191 mass chromatogram. In the TIC, peaks labeled with Arabic numbers refer to *n*-alkanes. Dipl: diploptene. 27β: 17β(H)-trinorhopane. 29ββ: 17β(H), 22β(H)-norhopane. 31αβ: 22R-17α(H), 22β(H)-homohopane. 30ββ: 17β(H), 22β(H)-hopane. 31ββ: 22R-17β(H), 22β(H)-homohopane. Of these, diploptene can be directly biosynthesized whereas the other structures are likely degradation products of biological hopanoids[1]. In the aliphatic fraction of peat extracts, a predominance with C_{31} αβ-homohopane is a characteristic of acidic peats with pH<6[2].

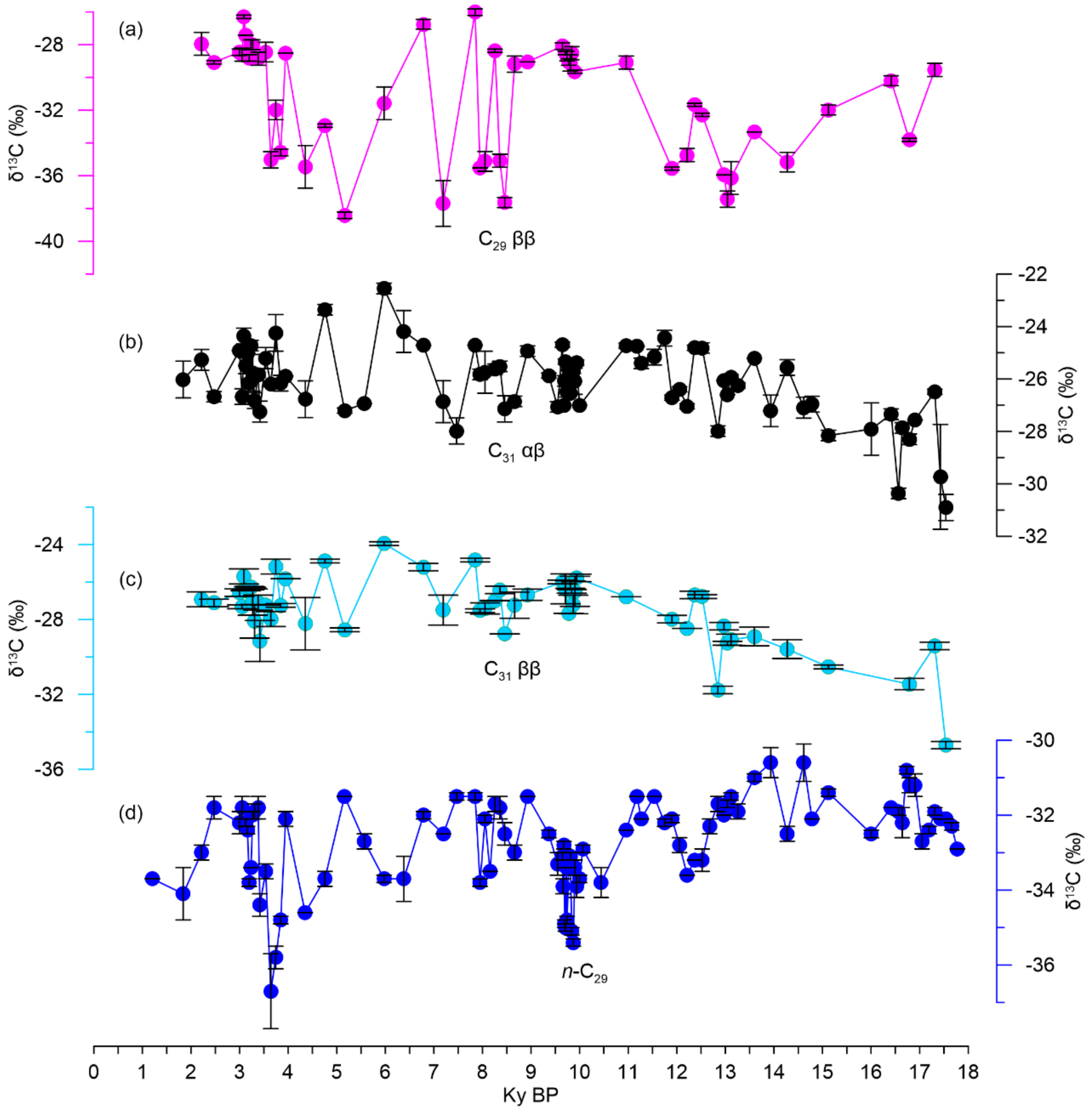

Supplementary Figure 4. Variations of the lipid $\delta^{13}C$ values in ZK-5 core. (a) C_{29} **ββ** hopane. (b) C_{31} **αβ** hopane. (c) C_{31} **ββ** hopane. (d) C_{29} *n*-alkane. Error bars represent 1 s.d. of replicate runs.

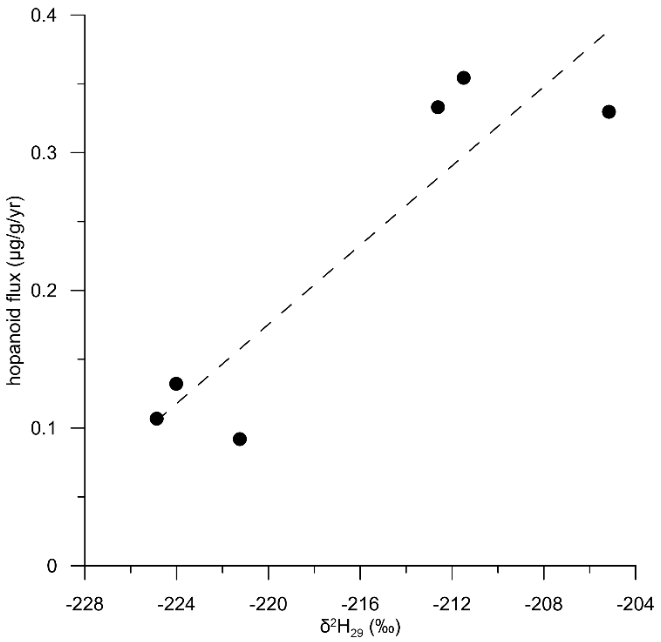

Supplementary Figure 5. Correlation between averaged *n*-C_{29} δ^2H values and the hopanoid flux. The correlation coefficient (r) between *n*-C_{29} δ^2H values and the hopanoid abundance is 0.26 (p= 0.05). However, hopanoid and *n*-alkane signals will not be directly correlated in a given horizon due to stratigraphic offsets arising from depth of production, i.e. during a dry and oxidizing interval, enhanced hopanoid production could occur in the peat subsurface. Consequently, the main text emphasizes the broad time interval of variation in all of these parameters. However, if we bundle intervals at 1-3ka, 3-5 ka, 5-7.2ka, 7.2-9.2 ka, 9.2-11.6 ka and 11.6-12.8 ka, then we observe a relatively strong correlation (r= 0.9), driven largely by data clustering into low hopanoid abundance/low δ^2H and high abundance/high δ^2H groups, thereby confirming the general interpretation in the main text.

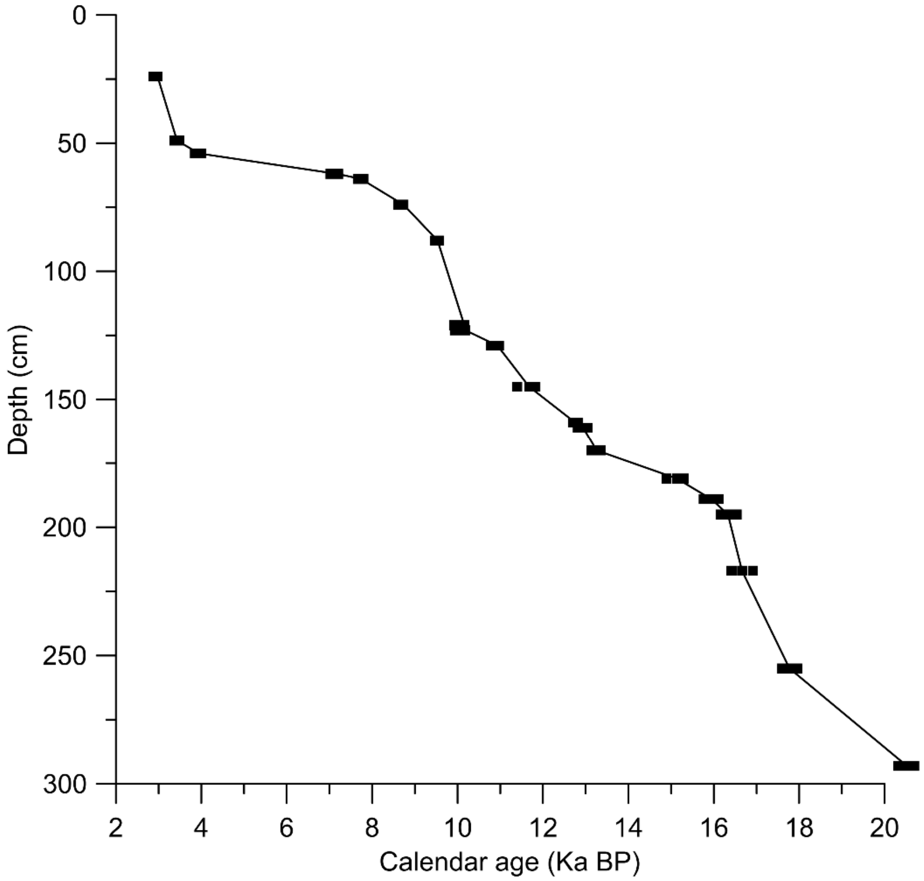

Supplementary Figure 6. Chronology of the ZK-5 core. The calendar ages were calibrated using the Clam package with 2 σ error intervals[3]. The left and right sides of the rectangles represent the lower and upper range of the calendar age, respectively.

Supplementary Table 1. **Hydrogen and oxygen isotope values of precipitation samples collected in Dajiuhu during June and July 2015**.

Day	δ^2H (‰)	δ^{18}O (‰)
2015-06-5	-80.1	-10.4
2015-06-16	-72.8	-10.2
2015-06-17(-1)[#]	-72.4/-72.7[##]	-10.5/-10.6
2015-06-17(-2)	-72.3	-10.6
2015-06-25	-85.6/-82.8	-11.4/-11.2
2015-07-03	-68.0	-8.8
2015-07-14	-102.1	-13.9
2015-07-15	-102.5/-103.5	-14.1/-13.9

[#]: two rainfall water samples were collected on 17th June 2015.

[##]: repeated measurement.

Supplementary Table 2. **Concentrations of *n*-alkanes (μg/g dry weight) and TOC content in the samples retrieved from the ZK-5 core**.

Age/ka	TOC/%	C_{21}	C_{22}	C_{23}	C_{24}	C_{25}	C_{26}	C_{27}	C_{28}	C_{29}	C_{30}	C_{31}	C_{32}	C_{33}
1.20	23.9	0.6	0.9	2.4	1.6	4.8	2.7	10.7	4.2	17.0	5.3	27.3	0.8	14.4
1.84	31.0	0.8	0.9	2.3	1.6	6.3	2.6	15.5	5.2	25.6	8.3	41.7	0.6	21.4
2.22	45.9	2.8	1.8	9.5	3.1	14.7	3.2	21.8	6.3	35.1	5.9	58.3	3.1	39.8
2.47	42.1	1.0	1.0	4.1	2.2	14.3	3.3	27.5	5.3	27.6	4.7	45.9	0.5	22.4
3.00	42.7	1.6	1.4	7.2	3.2	20.0	4.4	35.5	6.9	36.3	4.4	60.8	3.8	29.5
3.05	41.4	1.5	1.4	6.0	2.9	18.2	4.1	36.3	7.0	32.4	5.5	48.9	0.7	23.3
3.09	41.8	1.1	1.0	4.6	2.0	13.1	2.7	24.3	3.9	19.8	3.9	31.0	0.4	14.6
3.13	42.3	1.4	1.7	5.7	2.8	18.4	3.8	33.5	5.4	27.7	3.7	38.9	3.0	20.0
3.16	42.7	1.9	1.8	8.3	3.9	25.5	5.2	45.3	6.5	34.7	3.3	48.9	0.4	22.8
3.20	43.3	1.3	1.4	6.6	3.5	26.5	5.0	46.2	6.3	33.7	3.2	45.0	4.0	24.5
3.24	44.1	1.3	1.3	7.0	3.5	28.8	5.1	48.1	6.0	32.8	3.0	50.4	0.3	23.6
3.27	44.7	1.4	1.5	8.3	4.4	34.0	6.4	60.6	8.4	41.4	3.4	53.9	0.4	26.7
3.31	44.5	1.5	1.6	9.0	4.6	35.2	6.3	58.8	7.2	38.5	3.3	44.8	3.4	23.5
3.38	46.8	1.5	1.5	7.7	3.4	22.4	3.9	32.7	4.0	21.2	2.3	33.7	0.2	13.2
3.42	43.6	4.3	3.2	18.0	7.5	48.6	8.7	68.5	8.5	44.5	11.9	60.6	2.3	106.7
3.54	43.6	0.5	0.3	1.4	0.5	3.3	0.5	4.6	0.5	3.2	2.4	4.9	0.2	2.7
3.64	43.6	5.6	4.0	17.9	7.4	48.6	8.3	74.4	10.2	54.3	14.2	79.1	2.8	121.8
3.74	43.6	5.4	4.1	18.4	7.7	51.8	9.1	76.9	10.8	55.9	13.0	81.5	2.1	108.5
3.85	41.0	5.1	4.0	16.3	7.2	48.8	9.3	80.0	10.3	57.3	14.8	87.2	5.3	119.1
3.95	29.3	2.2	1.6	7.0	2.8	19.7	3.4	29.7	4.0	22.6	3.4	41.9	0.9	19.2
4.35	40.3	7.2	4.9	21.3	8.9	59.1	10.0	86.0	11.7	60.6	17.6	91.2	3.8	137.6
4.76	43.5	7.0	4.4	17.2	7.2	54.3	9.8	96.8	15.0	75.5	19.3	122.1	4.2	161.1
5.16	40.1	7.0	4.2	11.6	5.3	40.7	7.3	68.3	8.6	49.2	25.8	90.2	2.6	208.4

5.57	45.4	1.7	1.1	3.0	1.6	13.5	2.5	22.9	3.2	20.2	4.3	46.6	0.6	22.7
5.97	42.2	5.7	3.6	10.5	5.7	51.2	9.8	91.5	15.6	81.5	31.5	160.3	3.5	218.7
6.38	42.2	5.9	3.2	9.6	5.5	48.3	9.6	86.4	16.1	75.3	35.7	147.1	4.0	191.0
6.78	41.1	1.4	0.9	2.7	1.5	13.1	2.6	23.6	3.7	20.3	4.4	44.8	0.2	23.2
7.19	42.2	3.4	2.3	7.6	4.3	35.7	7.2	62.3	10.6	53.0	26.1	104.5	5.6	114.8
7.47	41.2	2.9	2.0	7.1	3.8	29.8	6.2	51.3	9.0	48.4	20.4	91.6	2.6	146.2
7.85	43.8	1.6	1.1	4.2	2.1	17.3	3.5	38.9	7.3	41.5	6.7	86.3	4.0	37.4
7.95	43.3	6.3	4.0	16.9	9.8	66.3	14.7	128.5	28.6	150.1	31.1	243.5	3.1	154.3
8.05	44.3	5.2	5.1	20.1	10.8	63.6	16.2	119.2	31.0	151.6	19.2	225.2	2.2	146.4
8.15	46.9	4.8	3.7	15.1	7.7	49.9	11.7	90.1	22.9	118.6	20.5	185.7	2.8	112.4
8.26	45.1	1.4	1.0	3.8	1.9	12.8	2.7	20.9	4.8	27.3	4.5	43.8	0.4	6.7
8.36	45.8	2.8	2.1	8.0	4.1	25.9	6.3	48.3	11.7	63.3	11.4	100.3	1.0	91.4
8.46	44.3	5.9	4.3	16.4	8.1	47.3	11.4	84.1	20.5	113.8	20.2	180.3	1.5	166.2
8.66	46.1	2.2	1.7	6.0	2.9	16.4	3.6	27.3	9.4	35.7	4.9	52.3	3.3	22.0
8.93	47.2	2.7	1.9	7.6	2.8	13.4	2.6	16.2	4.3	25.0	5.0	42.4	0.1	13.2
9.37	42.8	1.6	1.8	6.0	2.3	11.9	2.6	27.5	3.3	24.2	4.3	37.5	0.1	19.0
9.55	38.5	2.4	1.8	5.8	2.1	9.1	2.7	19.7	3.5	22.5	3.3	32.0	0.2	19.9
9.64	47.8	1.7	1.0	6.5	1.8	9.1	2.0	12.9	3.1	17.4	4.2	26.5	1.7	9.6
9.66	48.1	1.1	0.5	2.9	1.8	3.3	0.5	3.2	1.3	3.9	2.7	9.3	0.6	4.2
9.68	45.5	2.0	1.3	7.6	2.8	11.2	2.7	16.3	6.8	18.5	3.2	19.4	2.2	10.8
9.69	47.7	3.9	3.8	14.5	6.1	21.2	4.8	24.4	6.8	26.3	3.0	28.7	0.1	13.1
9.71	47.1	2.0	1.9	7.1	2.9	10.3	2.3	11.8	2.8	12.1	3.0	15.1	1.4	5.8
9.73		1.3	1.2	4.7	1.8	6.0	1.3	6.0	1.4	6.9	3.1	10.0	0.1	3.9
9.74	45.0	2.5	2.2	9.3	3.8	14.7	3.5	20.4	4.4	19.5	3.3	22.2	0.4	11.3
9.77	44.0	2.2	1.5	7.4	3.8	13.0	2.6	17.6	3.5	20.1	3.3	25.9	0.3	12.8

9.81	45.1	2.0	1.2	8.0	2.6	2.1	2.7	19.1	4.1	27.4	4.7	35.8	1.0	16.0
9.84	46.1	1.5	0.8	4.4	1.5	8.0	1.8	11.3	2.5	15.0	3.6	24.6	1.2	13.1
9.87	46.2	2.5	1.6	4.7	2.0	8.5	2.2	12.2	3.1	17.1	3.5	25.9	0.2	18.5
9.90	45.6	2.0	1.4	8.2	2.7	13.0	2.7	18.3	3.8	23.7	3.8	36.0	0.8	18.1
9.94	44.9	2.8	1.3	11.3	2.6	13.3	2.3	17.1	3.3	25.0	5.6	34.8	1.9	15.0
10.00	46.9	2.5	3.0	6.9	4.2	14.2	4.0	30.6	4.8	26.5	3.6	31.1	3.4	14.7
10.07	46.2	2.0	2.1	6.0	2.9	13.5	2.2	25.6	2.4	16.9	3.2	21.2	1.1	9.5
10.44	45.2	2.7	1.5	11.0	2.5	11.8	2.8	16.7	6.4	25.9	3.9	30.0	2.0	19.0
10.96	46.7	2.3	1.3	8.2	2.2	9.8	2.2	12.2	3.7	23.9	4.6	39.5	2.6	35.6
11.18	46.0	3.1	1.5	10.7	3.3	20.7	2.3	14.0	4.6	27.7	5.2	31.8	2.4	26.8
11.27	37.0	2.3	1.4	7.8	2.9	17.0	3.0	18.4	9.0	26.9	3.1	30.7	0.8	25.5
11.54	34.5	2.2	1.8	6.0	2.6	12.1	2.7	17.7	8.8	28.0	4.1	35.6	2.1	23.4
11.75	29.3	1.0	0.5	2.2	1.0	5.7	1.3	9.2	4.9	18.7	6.1	34.2	1.4	12.9
11.90	30.6	2.2	1.7	5.1	3.4	11.5	2.8	21.1	10.5	36.4	6.1	63.2	4.3	7.4
12.06	28.3	1.4	1.2	3.8	1.7	8.1	2.4	16.6	8.5	28.0	5.1	38.0	1.1	15.5
12.21	42.0	3.1	3.0	7.7	5.1	15.2	3.5	26.3	5.6	25.5	4.5	29.4	2.5	15.0
12.37	38.6	3.5	2.6	9.6	4.9	15.6	3.2	24.3	5.4	26.1	4.2	30.4	2.4	12.9
12.52	28.8	3.7	2.7	10.4	5.8	18.8	4.2	28.1	6.6	32.9	6.8	40.4	3.2	9.8
12.68	33.0	3.0	2.2	8.3	4.3	17.8	4.2	23.0	7.3	31.3	6.4	38.4	3.0	9.7
12.85	30.1	1.4	1.5	6.0	3.4	16.0	4.0	24.5	6.1	29.9	3.6	33.6	2.7	17.0
12.97	31.1	6.0	6.3	20.0	11.3	44.8	10.2	56.4	12.7	51.5	10.3	51.1	4.9	10.6
13.04	31.0	12.2	9.2	28.8	14.2	55.7	13.8	70.1	14.6	61.6	13.7	59.6	7.1	10.8
13.12	32.0	22.7	13.6	39.9	18.3	69.1	17.6	87.4	15.5	78.8	14.6	83.1	6.7	7.8
13.26	23.5	8.0	6.6	18.2	8.2	27.4	7.5	35.1	7.2	31.8	2.7	33.6	3.9	14.4
13.60	25.3	29.8	22.2	60.3	25.6	92.3	25.2	127.3	26.9	123.6	5.1	133.0	0.4	18.1

13.94	26.8	22.3	19.7	53.6	25.3	80.9	22.3	107.9	21.9	97.8	3.5	96.3	0.9	11.9
14.27	22.3	23.9	23.1	61.2	27.8	96.9	29.0	141.9	32.9	142.6	4.2	149.9	0.5	13.1
14.61	21.0	23.0	22.3	56.1	23.9	78.8	22.4	109.5	23.1	103.1	4.5	107.3	0.4	8.8
14.78	24.9	4.1	3.9	10.6	4.5	17.2	4.1	19.6	4.3	19.6	2.4	23.8	0.9	7.8
15.12	26.9	7.2	6.3	18.0	7.4	24.4	6.5	29.9	6.2	29.5	2.0	28.7	0.5	9.5
16.01	18.0	6.0	5.5	15.5	6.3	19.6	5.8	27.2	5.6	27.0	2.3	29.2	0.7	7.8
16.41	18.4	4.3	2.1	6.9	2.4	7.8	2.6	11.0	2.9	15.4	2.9	27.5	3.9	8.4
16.57	18.5	6.0	5.7	15.8	6.2	23.0	5.8	26.9	5.7	28.4	2.6	31.2	2.2	8.9
16.64	18.6	5.4	3.9	12.0	4.9	15.0	5.7	23.2	5.7	29.4	2.7	34.7	13.9	9.9
16.73	12.0	4.5	4.5	12.7	5.4	17.9	7.0	29.2	6.3	29.4	2.5	27.8	2.0	7.2
16.79	20.1	6.6	5.9	15.0	5.8	18.6	5.6	25.2	5.4	27.1	2.5	26.5	6.8	6.6
16.90	28.6	4.0	3.2	9.1	3.5	13.2	3.4	16.1	3.8	22.2	4.1	28.1	2.1	8.6
17.05	14.4	10.0	8.8	21.4	8.3	23.3	7.1	32.3	7.0	38.3	1.5	48.7	14.1	12.0
17.19	16.9	5.7	5.9	13.6	5.3	17.5	4.3	20.4	4.7	31.6	4.2	37.9	2.6	12.3
17.31	15.3	5.0	5.7	13.7	5.7	18.9	4.9	23.9	5.8	34.7	8.7	46.1	0.5	15.7
17.43	13.2	6.7	7.7	18.1	7.8	28.8	6.9	30.8	7.6	43.8	9.1	52.6	0.4	18.3
17.54	7.9	7.3	8.8	20.0	8.1	25.9	7.2	32.2	7.9	45.3	7.8	53.2	0.2	17.7
17.66	7.2	2.8	3.0	8.4	3.6	15.0	3.6	17.9	4.0	26.5	7.6	32.9	0.2	11.6

Supplementary Table 3. **The δ^2H and δ^{13}C values (‰) of C_{29} and C_{31} *n*-alkanes in ZK-5 core**. SD refers the standard deviation of replicate runs.

Age/ka BP	$\delta^{13}C_{29}$	SD	$\delta^{13}C_{31}$	SD	δ^2H_{29}	SD	δ^2H_{31}	SD
1.20	-33.7	0.0	-32.8	0.0	-218	2	-220	2
1.84	-34.1	0.7	-33.1	0.1	-231	7	-230	2
2.22	-33.0	0.2	-32.4	0.2	-226	2	-227	3
2.47	-31.8	0.3	-30.9	0.1	-222	2	-221	1
3.00	-32.2	0.3	-31.7	0.6	-223	1	-223	2
3.05	-31.8	0.3	-30.9	0.2	-222	0	-220	0
3.09	-32.5	0.3	-31.8	0.2				
3.13	-32.2	0.1	-31.2	0.1	-222	3	-220	2
3.16	-32.4	0.1	-31.4	0.0	-224	1	-223	1
3.20	-33.8	0.1	-32.0	0.2	-224	3	-218	2
3.24	-33.4	0.0	-32.6	0.1	-224	3	-219	6
3.27	-32.0	0.1	-31.6	0.0	-225	4	-221	1
3.31	-31.9	0.2	-30.9	0.0	-223	3	-218	1
3.38	-31.8	0.3	-31.4	0.1	-217	5	-210	0
3.42	-34.4	0.3	-32.2	0.2	-213	0	-210	1
3.54	-33.5	0.2	-33.0	0.3	-214	2	-230	1
3.64	-36.7	1.0	-33.5	0.4	-215	1	-212	2
3.74	-35.8	0.3	-33.5	0.1	-218	3	-214	1
3.85	-34.8	0.1	-32.7	0.2	-219	1	-217	2
3.95	-32.1	0.2	-31.2	0.1	-226	1	-223	0
4.35	-34.6	0.0	-32.8	0.1	-191	0	-203	2
4.76	-33.7	0.2	-32.3	0.3	-202	1	-215	1
5.16	-31.5	0.0	-30.5	0.1	-217	7	-217	7
5.57	-32.7	0.2	-32.1	0.2	-233	0	-228	4
5.97	-33.7	0.1	-32.6	0.1	-192	7	-211	2
6.38	-33.7	0.6	-32.3	0.0	-210	4	-222	0
6.78	-32.0	0.1	-31.5	0.0	-225	0	-229	0
7.19	-32.5	0.0	-31.4	0.2	-204	2	-219	3
7.47	-31.5	0.1	-30.3	0.2	-217	0	-224	0
7.85	-31.5	0.1	-30.5	0.1	-228	2	-236	1
7.95	-33.8	0.1	-32.3	0.2	-232	1	-232	2
8.05	-32.1	0.1	-31.4	0.2	-231	1	-231	1
8.15	-33.5	0.0	-32.8	0.1	-231	2	-228	0
8.26	-31.7	0.2	-30.8	0.0				
8.36	-31.8	0.3	-31.0	0.1	-230	1	-229	0
8.46	-32.5	0.3	-31.3	0.0	-227	1	-223	1
8.66	-33.0	0.2	-32.0	0.2	-225		-224	
8.93	-31.5	0.0	-31.0	0.0	-228	1	-229	0
9.37	-32.5	0.1	-31.0	0.1				

9.55	-33.3	0.3	-32.2	0.1	-219	5	-219	1
9.64	-33.1	0.1	-32.8	0.1	-233	3	-230	1
9.66	-33.9	0.2	-32.3	0.2	-222	1	-220	1
9.68	-32.8	0.1	-32.4	0.1	-231	3	-223	6
9.69	-34.9	0.1	-32.8	0.5	-221		-241	
9.71	-35.0	0.1	-34.8	0.1	-226	4	-230	9
9.73	-33.4	0.0	-32.7	0.1				
9.74	-34.8	0.1	-32.4	0.1	-221	3	-215	5
9.77	-33.4	0.0	-32.8	0.0	-229	6	-226	3
9.81	-33.1	0.1	-32.6	0.1	-226	0	-230	1
9.84	-35.1	0.1	-34.1	0.3	-222	2	-224	8
9.87	-35.4	0.1	-33.9	0.1	-221	0	-207	0
9.90	-33.4	0.2	-32.8	0.1	-223	0	-233	2
9.94	-33.9	0.3	-32.9	0.6	-229	2	-232	3
10.00	-33.7	0.1	-33.3	0.1	-224	2	-219	1
10.07	-32.9	0.1	-31.6	0.1	-217	6	-206	5
10.44	-33.8	0.4	-33.6	0.1	-207	0	-206	1
10.96	-32.4	0.0	-32.1	0.0	-214	0	-206	0
11.18	-31.5	0.0	-31.0	0.1	-217	1	-207	3
11.27	-32.1	0.0	-30.3	0.8	-211	0	-205	1
11.54	-31.5	0.0	-31.4	0.1	-221	3	-219	2
11.75	-32.2	0.1	-32.0	0.2	-218	1	-214	0
11.90	-32.1	0.1	-32.9	0.1	-219	1	-225	1
12.06	-32.8	0.2	-31.5	0.1	-226	3	-221	1
12.21	-33.6	0.0	-32.2	0.1	-222	0	-224	0
12.37	-33.2	0.0	-32.0	0.1	-221	1	-217	2
12.52	-33.2	0.3	-32.0	0.2	-219	1	-218	1
12.68	-32.3	0.2	-32.0	0.0	-219	1	-216	2
12.85	-31.7	0.2	-31.7	0.3	-225	5	-226	8
12.97	-32.0	0.0	-31.7	0.0	-220	2	-212	2
13.04	-31.7	0.1	-31.6	0.2	-214	0	-209	1
13.12	-31.5	0.1	-31.4	0.1	-209	0	-207	1
13.26	-31.9	0.2	-32.4	0.1	-205	1	-209	5
13.60	-31.0	0.1	-30.8	0.1	-206	2	-202	2
13.94	-30.6	0.4	-30.8	0.3	-205	1	-203	0
14.27	-32.5	0.2	-31.7	0.1	-203	0	-204	1
14.61	-30.6	0.5	-30.8	0.3	-201	2	-193	5
14.78	-32.1	0.0	-31.5	0.0	-198	0	-197	2
15.12	-31.4	0.1	-32.2	0.1	-189	6	-191	7
16.01	-32.5	0.1	-32.9	0.1	-193	5	-202	2
16.41	-31.8	0.0	-31.7	0.0	-206	1	-207	3
16.57	-31.9	0.1	-32.0	0.1	-188	4	-194	2

16.64	-32.2	0.4	-32.7	0.1	-193	3	-208	2
16.73	-30.8	0.1	-31.8	0.1	-190	4	-218	3
16.79	-31.2	0.2	-31.2	0.1	-206	1	-210	0
16.90	-31.2	0.3	-31.2	0.2	-210	5	-216	8
17.05	-32.7	0.2	-32.6	0.2	-201	0	-207	2
17.19	-32.4	0.1	-32.0	0.1	-217	1	-219	0
17.31	-31.9	0.1	-31.5	0.0				
17.43	-32.1	0.0	-31.4	0.0	-203	1	-199	0
17.54	-32.1	0.0	-31.7	0.2	-190	1	-197	2
17.66	-32.3	0.1	-31.5	0.1	-205	1	-205	1

Supplementary Table 4. **The δ^{13}C values (‰) of hopanes in the samples retrieved from the ZK-5 core**. SD refers the standard deviation of replicate runs.

age/ka BP	δ^{13}C$_{29\beta\beta}$	SD	δ^{13}C$_{31\alpha\beta}$	SD	δ^{13}C$_{30\beta\beta}$	SD	δ^{13}C$_{31\beta\beta}$	SD
1.84		0.5	-26.0	0.7				1.9
2.22	-28.0	0.7	-25.3	0.4	-26.7	0.3	-26.9	0.4
2.47	-29.1	0.1	-26.7	0.2	-28.3	0.3	-27.1	0.2
3.00	-28.5	0.2	-24.9	0.0	-25.8	1.2	-26.6	0.2
3.05	-28.6	0.4	-26.7	0.3		0.3	-27.3	0.1
3.09	-26.3	0.1	-24.4	0.3	-30.3	0.3	-25.7	0.4
3.13	-27.4	0.0	-25.5	0.3	-30.7	0.2	-26.5	0.0
3.16			-25.0	0.0				3.6
3.20	-28.8	0.2	-26.2	0.1	-27.5	0.2	-26.3	0.1
3.24	-28.0	0.3	-24.7	0.2	-26.7	0.1	-26.3	0.0
3.27	-28.0	0.3	-25.8	0.7	-26.2	2.1	-27.1	0.7
3.31		0.4	-26.8	0.3		1.6	-28.1	0.9
3.38	-28.9	0.4	-25.8	0.1	-27.0	0.3	-27.1	0.4
3.42		0.7	-27.2	0.4			-29.1	1.1
3.54	-28.5	0.6	-25.2	0.4		3.8	-27.2	0.0
3.64	-35.0	0.5	-26.2	0.1	-35.8	0.2	-28.0	0.4
3.74	-32.0	0.6	-24.2	0.7	-34.4	0.0	-25.2	0.4
3.85	-34.6	0.2	-26.2	0.3	-35.6	1.1	-27.2	0.1
3.95	-28.5	0.0	-25.9	0.0	-28.9	2.0	-25.8	0.0
4.35	-35.5	1.3	-26.8	0.7	-35.6	0.2	-28.2	1.4
4.76	-32.9	0.1	-23.4	0.2	-34.5	0.4	-24.9	0.1
5.16	-38.4	0.2	-27.2	0.1	-37.9	0.0	-28.5	0.1
5.57			-26.9	0.0				
5.97	-31.6	1.0	-22.5	0.2	-35.9	0.2	-23.9	0.1
6.38		0.2	-24.2	0.8		0.5		0.5
6.78	-26.8	0.3	-24.7	0.0		0.7	-25.2	0.2
7.19	-37.7	1.4	-26.9	0.8	-37.4	0.6	-27.5	0.8
7.47		0.7	-28.0	0.5		8.8		0.1
7.85	-26.0	0.2	-24.7	0.0	-28.1	0.5	-24.8	0.1
7.95	-35.5	0.0	-25.8	0.2	-36.8	0.1	-27.5	0.1
8.05	-35.1	0.6	-25.7	0.8	-36.8	0.4	-27.4	0.3
8.26	-28.4	0.1	-25.6	0.2		0.4	-27.0	0.0
8.36	-35.1	0.4	-25.5	0.2	-37.8	0.4	-26.4	0.2
8.46	-37.6	0.3	-27.1	0.5	-40.7	5.3	-28.8	0.0
8.66	-29.2	0.5	-26.9	0.2		1.7	-27.2	0.7
8.93	-29.1	0.0	-24.9	0.2		0.2	-26.7	0.3
9.37		0.1	-25.9	0.1				5.6
9.55		0.6	-27.1	0.2		0.3		3.6
9.64	-28.1	0.2	-24.7	0.1	-26.1	0.1	-26.0	0.1

9.68			-27.0	0.0				
9.69		0.0	-26.1	0.2		0.1		0.2
9.71	-28.5	0.1	-25.3	0.1		0.8	-26.4	0.8
9.74	-28.7	0.3	-26.5	0.2				0.5
9.77	-29.1	0.2	-25.7	0.0	-26.7	0.2	-27.7	0.0
9.81	-29.1	0.5	-26.6	0.0		0.1	-26.1	0.2
9.84	-28.5	0.4	-25.7	0.0		0.6	-26.5	0.1
9.87		1.6	-25.7	0.1		0.9	-27.2	0.5
9.90	-29.7	0.1	-26.1	0.5	-27.9	0.5	-26.5	0.8
9.94		0.4	-25.4	0.1		0.4	-25.8	0.2
10.00		0.5	-27.0	0.0		0.5		0.8
10.96	-29.1	0.4	-24.7	0.1		0.1	-26.8	0.0
11.18		0.0	-24.8	0.0				0.6
11.27		0.3	-25.4	0.2		0.0		0.6
11.54		1.5	-25.2	0.3		0.2		1.0
11.75		1.0	-24.4	0.3				0.0
11.90	-35.6	0.1	-26.7	0.1	-39.6	0.6	-28.0	0.2
12.06		0.1	-26.4	0.1		0.0		2.5
12.21	-34.7	0.4	-27.0	0.1		0.4	-28.5	0.0
12.37	-31.7	0.1	-24.8	0.1	-36.3	0.1	-26.7	0.2
12.52	-32.3	0.1	-24.8	0.2	-36.5	0.2	-26.8	0.1
12.85		0.3	-28.0	0.2		0.0	-31.8	0.2
12.97	-35.9	0.0	-26.1	0.0	-37.2	0.3	-28.4	0.2
13.04	-37.4	0.5	-26.6	0.0	-37.2	2.3	-29.3	0.1
13.12	-36.1	1.0	-25.9	0.1	-36.0	0.2	-29.1	0.3
13.26		0.5	-26.3	0.1		0.5		0.0
13.60	-33.3	0.0	-25.2	0.0	-35.7	0.7	-28.9	0.5
13.94		0.7	-27.2	0.6				0.6
14.27	-35.2	0.6	-25.6	0.3	-36.5	0.7	-29.6	0.5
14.61		1.0	-27.1	0.4		0.1		0.1
14.78		0.3	-27.0	0.3		1.0		0.3
15.12	-32.0	0.3	-28.2	0.2		0.1	-30.5	0.1
16.01		0.4	-27.9	1.0		0.1		0.1
16.41	-30.2	0.3	-27.3	0.2		0.1		0.1
16.57		1.1	-30.4	0.2		0.3		0.2
16.64			-27.9	0.2				
16.79	-33.8	0.1	-28.3	0.2	-29.1	0.2	-31.4	0.3
16.90		0.5	-27.6	0.0		0.4		0.5
17.31	-29.5	0.4	-26.5	0.1	-28.3	0.1	-29.4	0.2
17.43		0.2	-29.7	2.0		0.4		0.4
17.54		1.6	-30.9	0.5		0.8	-34.7	0.2

Supplementary Table 5. **Results of ^{14}C AMS dating in ZK-5 core**.

Lab code	AMS code	Depth/cm	^{14}C age/error	Cal age (BP*)
DJH-24	432094	24	2860±30	3066–2917
DJH-49	369149	49	3220±30	3484–3375
DJH-54	432095	54	3630±30	3993–3857
DJH-62	467928	62	6250±30	7057–7027
DJH-64	432096	64	6920±30	7800–7681
DJH-74	385688	74	7920±30	8798–8627
DJH-88	381457	88	8560±40	9561–9478
DJH-121	369150	121	8980±50	10238–10116
DJH-123	381458	123	8990±40	10239–10128
DJH-129	425598	129	9620±30	10975–10787
DJH-145	425481	145	10090±40	11830–11402
DJH-159	425599	159	10890±40	12819–12700
DJH-161	393241	161	11070±30	13047–12817
DJH-170	369151	170	11420±40	13357–13145
DJH-181	381459	181	12700±50	15304–14895
DJH-189	425600	189	13260±40	16109–15763
DJH-195	390115	195	13560±40	16539–16164
DJH-217	421870	217	13780±50	16918–16415
DJH-255	381460	255	14590±50	17952–17598
DJH-293	425601	293	17010±50	20692–20323

* BP: before the present (1950 AD).

Supplementary Note 1

Evaporation difference between the Dajiuhu peatland and Sanbao cave

Compared with the Dajiuhu peatland, the Sanbao cave is less affected by evaporation. This cave is located at a relatively high elevation (1900 m above the sea level), with deeper overlying soil and dense forest cover. Here the precipitation has a relatively short residence time in the uppermost soil, which might escape, to some extent, the influence of evaporation. Further evidence that evaporation shows little effect on stalagmite $\delta^{18}O$ in Sanbao site is that Holocene stalagmite $\delta^{18}O$ sequences show high consistency from southwest China to northeast China, spanning a distance of >2000 km and encompassing quite different climate zones[4].

Supplementary References:

1. Talbot, H.M., Bischoff, J., Inglis, G.N., Collinson, M.E., Pancost, R.D. Polyfunctionalised bio- and geohopanoids in the Eocene Cobham Lignite. *Org. Geochem.* **96**, 77-92 (2016).
2. Huang, X., Meyers, P.A., Xue, J., Gong, L., Wang, X., Xie, S. Environmental factors affecting the low temperature isomerization of homohopanes in acidic peat deposits, central China. *Geochim. Cosmochim. Acta* **154**, 212-228 (2015).
3. Blaauw, M. Methods and code for 'classical' age-modelling of radiocarbon sequences. *Quarter. Geochronol.* **5**, 512-518 (2010).
4. Liu, Z., Wen, X., Brady, E.C., Otto-Bliesner, B., Yu, G., Lu, H., Cheng, H., Wang, Y., Zheng, W., Ding, Y., Edwards, R.L., Cheng, J., Liu, W., Yang, H. Chinese cave records and the East Asia summer monsoon. *Quat. Sci. Rev.* **83**, 115-128 (2014).
